# Genetic variation reveals the influence of steroid hormones on the risk of retinal neurodegenerative diseases

**DOI:** 10.3389/fendo.2022.1088557

**Published:** 2023-01-10

**Authors:** Kangcheng Liu, Huimin Fan, Hanying Hu, Yanhua Cheng, Jingying Liu, Zhipeng You

**Affiliations:** Jiangxi Province Division of National Clinical Research Center for Ocular Diseases, Jiangxi Clinical Research Center for Ophthalmic Disease, Jiangxi Research Institute of Ophthalmology and Visual Science, Affiliated Eye Hospital of Nanchang University, Nanchang, China

**Keywords:** steroid hormones, retinal neurodegenerative disease, Mendelian randomization, causality, glaucoma

## Abstract

It is difficult to get evidence from randomized trials of a causal relationship between steroid hormones produced by the adrenal gland and gonad and retinal neurodegenerative disorders (RND). In this study, genetic variations of aldosterone (Aldo), androstenedione (A4), progesterone (P4), hydroxyprogesterone (17-OHP), and testosterone/17β-estradiol (T/E2) were obtained from genome-wide association studies as instrumental variables. Mendelian randomization (MR) analysis was used to assess the impact on the risk of RND, including glaucoma (8,591 cases and 210,201 controls), diabetic retinopathy (DR, 14,584 cases and 202,082 controls) and age-related macular degeneration (AMD, 14,034 cases and 91,214 controls). As the main method, inverse variance weighted results suggest that the increased glaucoma risk was affected by T/E2 (OR = 1.11, 95% CI, 1.01–1.22, *P* = 0.03), which was further validated by other methods (*P_WM_* = 0.03, *P_MLE_* = 0.03, *P_MR-RAPS_*_=_ 0.03). In the replicated stage, the causal relationship between T/E2 and glaucoma was verified based on the MRC-IEU consortium (*P* = 0.04). No impact of Aldo, A4, P4, 17-OHP, and T/E2 was observed for the risk of DR (*P* > 0.05) and AMD (*P* > 0.05). The heterogeneity test (*P* > 0.05) and pleiotropy test (*P* > 0.05) verified the robustness of the results. Our results suggest that T/E2 has a suggestive effect on the glaucoma risk. However, the genetic evidence based on a large sample does not support the effect of steroid hormones on DR and AMD risk. Further studies are vital to assess the possibility of steroid hormones as targets for prevention and treatment.

## 1 Introduction

Steroid hormones, including aldosterone (Aldo), androstenedione (A4), progesterone (P4), hydroxyprogesterone (17-OHP), testosterone (T), and 17β-estradiol (E2), are produced primarily by the adrenal gland and gonads (1). As regulators of various physiological processes, their biological synthesis originates from cholesterol (2). 17-OHP is transformed from progesterone into A4, T, and Aldo. Moreover, androgens (such as A4 and T) are precursors for E2. The levels of these steroid hormones are in dynamic balance in the body and affect the body’s inflammation, metabolism, cell proliferation, and other physiological activities. When the adrenal gland or gonad is in a disease state (such as primary aldosteronism (PA), prostate cancer (PC), or ovarian tumor), steroid hormone levels will change significantly and further affect other organs (such as the heart) of the body.

Notably, steroid hormone receptors have been found throughout eye structures, including the retina (3, 4). These findings revealed that steroid hormones could likely influence visual processing and retinal neurodegenerative diseases (RND) risk. Some studies believe that changes in steroid hormone homeostasis will damage the retina, leading to glaucoma, age-related macular degeneration (AMD), diabetic retinopathy (DR), and other RND (5–7). The study of the rat model shows that Aldo can affect the retinal ischemic damage caused by glaucoma through the Renin-Angiotensin-Aldo System (8, 9). After systemic administration of Aldo, Nitta et al. (10) found progressive loss of retinal ganglion cells (RGCs) without elevated intraocular pressure (IOP), which means that PA will increase the glaucoma risk. Ohshima et al. compared 137 glaucoma patients with PA and 177 controls and found no difference in the prevalence of glaucoma optic disc appearance between the two groups (11). Hao et al. found that E2 can protect RGCs under a high glucose environment through the mitochondrial pathway (12). However, Siddiqui et al.’s study on 255 subjects suggested that E2 was not related to DR risk (13). Lin et al.’s cohort study found that elevated androgen levels in PC patients increased the AMD risk (14). POLA study monitored several steroid hormones in serum and found no correlation between T, E2, and AMD. From these contradictory results, it can be found that the steroid hormones’ effect on RND is still unclear. Clarifying the causal relationship between the steroid hormones and RND helps define the strategic approach of steroid hormone benefits, potentially valuable for RND.

However, due to the complexity of the steroid hormone system, many confounders affect the evaluation of randomized controlled studies. It is difficult for researchers to determine the effect of a single steroid hormone on the RND risk. To obtain more reliable results with large samples, the Mendelian randomization (MR) study provides an alternative method to explore the influence of various factors on RND risk through genetic variation (15, 16). M. Verbanck et al. used MR to analyze the influence of testosterone on prostate cancer, hypertension, and other diseases (17). Pott et al. (18) also found the sex-specific causal networks of steroid hormones and obesity through MR research. These studies provide additional possibilities for exploring the effects of steroid hormones on disease risk.

Therefore, to better explore the role of steroid hormones on the risk of RND, we performed a two-sample MR study in which we simultaneously obtained four different instrumental variables (IVs) of steroid hormones from genome-wide association studies (GWASs) of large samples. Through exploring causality, we hope to increase the understanding of the risk of steroid hormones affecting RND. Moreover, it provides a more theoretical basis for reducing the RND risk caused by changes in steroid hormone levels.

## 2 Methods

### 2.1 Design of the two-sample MR study

To investigate the effect of steroid hormone levels on the risk of RND (glaucoma, DR, and AMD), we conducted an MR study. Considering the winners’ curses and weak instruments, we chose a two-sample MR instead of a one-sample MR (19). According to Mendel’s laws of inheritance, single nucleotide polymorphisms (SNPs) are inherited independently and circumvented reverse causality. In addition, this two-sample MR study follows the MR-STROBE guidance (20).

### 2.2 Exposure: Genetic predictors for steroid hormones

In our study, steroid hormones mainly included Aldo, A4, P4, 17-OHP, and T/E2. Single nucleotide polymorphisms (SNPs) predicting levels of five steroid hormones were obtained from the GWAS by Pott et al. (18). This GWAS used data from LIFE-Adult (males/females: 863/618) (21) and LIFE-Heart (males/females: 1357/711) (22). In LIFE-Adult and LIFE-Heart, A4, P4, and 17-OHP were measured by liquid chromatography-tandem mass spectrometry (LC—MSMS). For T and E2, the electrochemiluminescence immunoassay was used for measurement in LIFE-Adult, and LC—MSMS was used for measurement in LIFE-Heart. At the same time, this GWAS based on two Life studies has adjusted age, sex, and log-transformed BMI and was imputed using 1000 Genomes (Phase 3) reference panel.

### 2.3 Outcome: summary-level data of RND

To explore the influence of steroid hormones on RND risk, we selected glaucoma, DR, and AMD as the main subjects in the discovery stage. The summary-level data of glaucoma and DR were obtained from FinnGen biobank (218,792 subjects; browser: r5.finngen.fi) (23). The GWASs from FinnGen biobank analyzed 16,962,023 variables and adjusted age, sex, genetic relatedness, genotyping batch, and first 10 principal components (PCs). Detailed information on glaucoma and DR on the GWASs is provided in **Table 1**. The summary-level data of AMD were obtained from the GWAS by Winkler et al. (24). This GWAS included 14,034 AMD patients and 91,214 controls through 11 data sources (**Table 1**). All data sources of the GWAS by Winkler et al. were processed through a standardized quality-control pipeline (25) and adjusted age, population stratification, DNA source, and two PCs.

**Table 1:** GWAS samples of retinal neurodegenerative diseases used in MR study.

Stage	Outcome	Cases	Controls	Population	Reference
Discovery	glaucoma	8,591	210,201	European	FinnGen biobank (22)
Discovery	DR	14,584	202,082	European	FinnGen biobank (22)
Discovery	AMD	14,034	91,214	European	Winkler et al (23)
Replicated	glaucoma	4,737	458,196	European	MRC-IEU consortium (25)

DR, Diabetic retinopathy; AMD, Age-related macular degeneration.

In the replicated stage, we chose the largest sample size, GWAS (ID: ukb-b-8398), as the replication outcome of glaucoma. Based on the MRC-IEU consortium (26), this GWAS analyzed 9,851,867 SNPs from 150,642 European samples. This GWAS was obtained from the MR base database (27), which contains 4,737 glaucoma cases and 458,196 controls (as of October 31, 2022) (**Table 1**).

### 2.4 IVs selection and assumption

**Figure 1:**
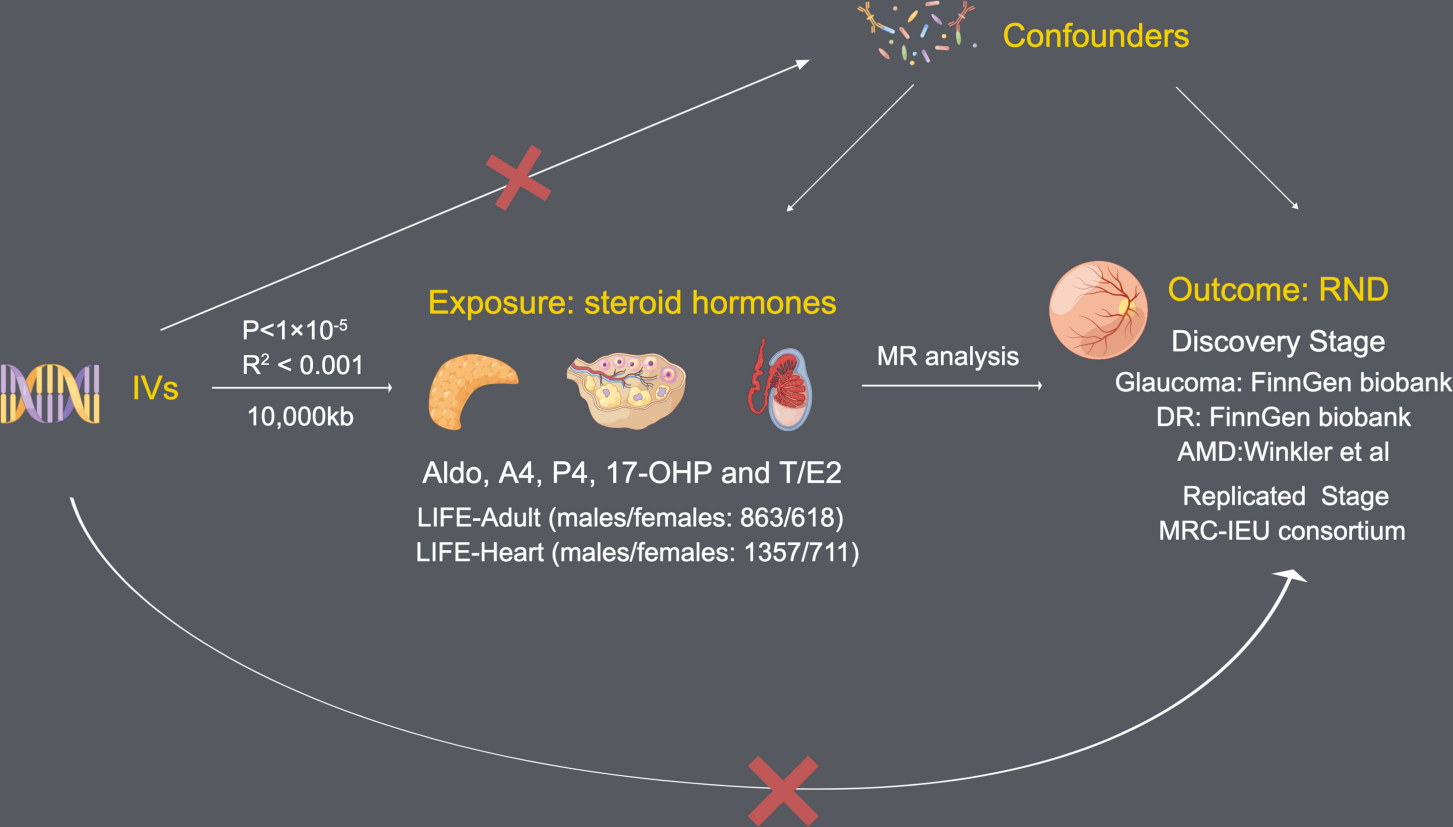
Assumptions of Mendelian randomization analysis between steroid hormone and RND.

To obtain reliable results, MR analysis needs to satisfy the following three assumptions (**Figure 1**): (1) Each SNP as IV is associated with each steroid hormone; (2) All IVs that passed quality selection should not be associated with confounders; (3) The effects of the IVs on the risk of each steroid hormone are only mediated by RND.

Since there are too few IVs obtained by selecting the threshold of 5×10^-8^ (genome-wide significance), *P* < 1×10^-5^ (locus-wide significance) is selected as the threshold to obtain higher statistical power for obtaining IVs, similar to other MR studies, this threshold is acceptable (28). A threshold (R^2^ < 0.001) and clumping distance (10,000 kb) were set for IVs to attenuate linkage disequilibrium (LD) by referring to the European-based 1,000 Genome Projects. Remove palindromic SNPs and use MR-Steiger filtering (29) to clarify the causality direction of each IV between steroid hormones and RND.

### 2.5 Methods of MR analysis

#### 2.5.1 Causal effect estimation

MR analysis of only one SNP was performed using the Wald ratio (WR) method. For causal estimation of multiple SNPs, inverse variance weighted (IVW) is the main evaluation method for MR analysis (30). At the same time, four additional methods are used to verify the results of IVW with causality between steroid hormones and RND: (1) MR-Egger (31): Even if most IVs have pleiotropy, it can provide effective estimates. However, compared with the other 4 methods, the causal estimates of MR-Egger may be biased; (2) Weighted median (32): The weighted median of the WR estimate is calculated to obtain a relatively robust causal estimate; (3) Maximum likelihood estimator (MLE) (33): the results assume the linear correlation of RND and each steroid hormone with jointly normal distribution and allow for uncertainty in both gene–steroid hormone and gene–RND associations; (4) MR robust adjusted profile score (MR-RAPS) (34): Even if there are weak IVs, the method can also provide robust causal estimates.

#### 2.5.2 Sensitivity analysis

MR Egger expression and MR-PRESSO tested the pleiotropy of MR results. Moreover, MR-PRESSO can also be used to remove outliers (35). Cochrane’s Q method tested the heterogeneity of MR results between steroid hormones and RND. At the same time, SNPs were deleted one by one by using the Leave-one-out method to evaluate whether they drove the causal effect between steroid hormones and RND.

### 2.6 Statistical analysis

All statistical analyses were completed in R software environment of version 2.22. R packages “TwoSampleMR” and “MR-PRESSO” were used to analyze the causality between steroid hormones and RND. F statistics < 10 is considered a weak IV (F = $\frac{R^{2}\left(n-k-1\right)}{k\left(1-R^{2}\right)}$ ; R^2^: GM taxa variance explained by SNPs; n: sample size; k: the number of included IVs) (36). The effect estimates of IVs predicted steroid hormones on RND were presented as odds ratio (OR) with a 95% confidence interval (CI). *P* < 0.05 was considered suggestive of significance and a potential causal effect.

## 3 Results

### 3.1 Causal associations between steroid hormones and glaucoma (discovery stage)

After data harmonization of GWAS data, 17 SNPs related to Aldo, 16 SNP s related to A4, 28 SNPs related to P4, 11 SNPs related to 17-OHP, and 15 SNPs related to T/E2 were used as IVs for MR analysis (**Supplementary Table S1**). All IVs are strong instruments (F, 19.52 to 85.15) (**Supplementary Table S1**).

**Figure 2:**
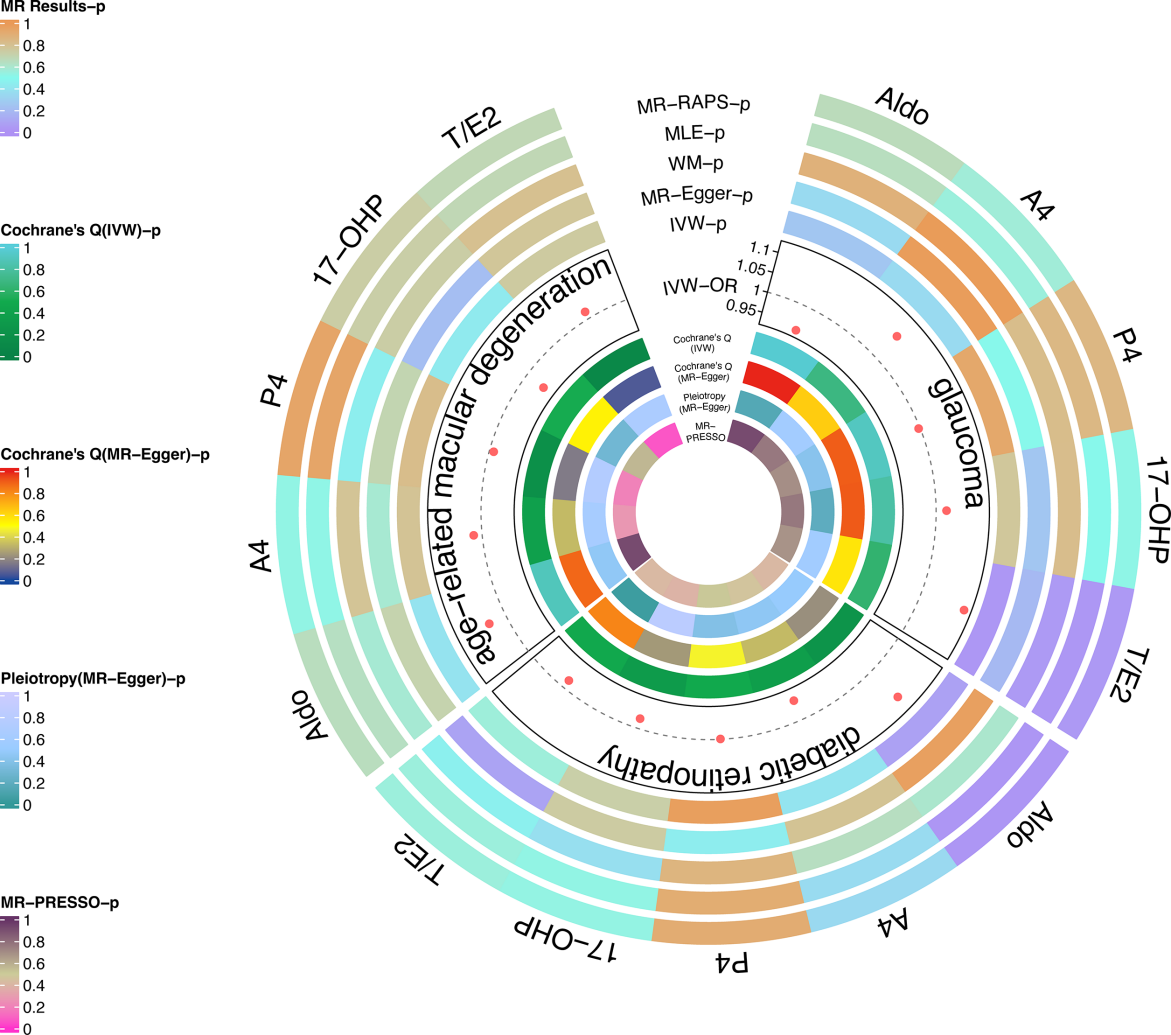
Causal analysis of steroid hormone and RND based on MR analyses and sensitivity analyses in the discovery stage.

As the most important method, IVW results found that the increased risk of glaucoma was affected by T/E2 (OR = 1.11, 95% CI, 1.01–1.22, *P* = 0.03) (**Figure 2** and **Table 2**). The results of WM (*P* = 0.03), MLE (*P* = 0.03), and MR-RAPS (*P* = 0.03) also verified this discovery (**Figure 2** and **Table 2**). The results of Aldo (*P_IVW_* = 0.25, *P_MR-Egge_*_r_ = 0.35, *P_WM_* = 0.88, *P_MLE_* = 0.65, *P_MR-RAPS_* = 0.66), A4 (*P_IVW_* = 0.35, *P_MR-Egge_*_r_ = 0.98, *P_WM_* = 0.99, *P_MLE_* = 0.55, *P_MR-RAPS_* = 0.57), P4 (*P_IVW_* = 0.93, *P_MR-Egge_*_r_ = 0.50, *P_WM_* = 0.82, *P_MLE_* = 0.85, *P_MR-RAPS_* = 0.85) and 17-OHP (*P_IVW_* = 0.77, *P_MR-Egge_*_r_ = 0.25, *P_WM_* = 0.82, *P_MLE_* = 0.51, *P_MR-RAPS_* = 0.52) did not suggest a significant causal relationship with glaucoma (**Figure 2** and **Table 2**).

**Table 2:** MR results and sensitivity analyses between steroid hormones and glaucoma.

	*P* (MR results)					*P* (Cochrane's Q)		*P* (Pleiotropy)	
	IVW	MR-Egger	WM	MLE	MR-RAPS	IVW	MR-Egger	MR-Egger regression	MR-PRESSO
Discovery stage									
Aldo	0.25	0.35	0.88	0.65	0.66	0.97	0.99	0.14	0.918
A4	0.35	0.98	0.99	0.55	0.57	0.68	0.64	0.59	0.767
P4	0.93	0.51	0.82	0.85	0.85	0.91	0.91	0.41	0.698
17-OHP	0.77	0.25	0.82	0.51	0.52	0.82	0.91	0.19	0.769
T/E2	0.03	0.19	0.04	0.03	0.03	0.62	0.57	0.58	0.679
Replicated stage									
Aldo	0.93	0.20	0.67	0.75	0.76	0.81	0.95	0.20	0.735
A4	0.35	0.75	0.80	0.32	0.35	0.87	0.81	0.66	0.935
P4	0.17	0.16	0.06	0.69	0.67	0.17	0.47	0.11	0.057
17-OHP	0.43	0.45	0.07	0.55	0.55	0.09	0.08	0.49	0.133
T/E2	0.04	0.44	0.11	0.04	0.05	0.65	0.77	0.29	0.675

MR, Mendelian randomization; IVW, Inverse variance weighted; WM, Weighted median; MLE, maximum likelihood estimator; MR-RAPS, MR robust adjusted profile score; Aldo, aldosterone; A4, androstenedione; P4, progesterone; 17-OHP, hydroxyprogesterone; T/E, Testosterone_Estradiol_Ratio.

The heterogeneity test used Cochrane’s Q method to confirm that there was no heterogeneity in MR results (IVW: all *P* > 0.62; MR-Egger: all *P* > 0.56) (**Figure 2** and **Table 2**). MR-Egger regression confirmed that the results did not have level pleiotropy (all *P* > 0.14) and was further verified by MR-PRESSO (all *P*> 0.67) (**Figure 2** and **Table 2**). More details are shown in **Supplementary Table S2**. Meanwhile, there was no significant difference in causal estimations of each steroid hormone on glaucoma by the leave-one-out analysis (**Supplementary Figure S1**).

### 3.2 Causal associations between steroid hormones and DR (discovery stage)

After quality control, the same number of SNPs as glaucoma was used as strong IVs (F, 19.52 to 85.15) for MR analysis (**Supplementary Table 1**).

The results of MLE (OR = 1.10, 95% CI, 1.05–1.14, *P* = 0.02) and MR-RAPs (OR = 1.10, 95% CI, 1.05–1.15, *P* = 0.02) found that Aldo had a potential causal relationship with cataract (**Figure 2** and **Table 3**). The main results for IVW were close to significant differences (*P* = 0.07) (**Figure 2** and **Table 3**). Heterogeneity tests (IVW: *P* = 0.23; MR-Egger: *P* = 0.21) and pleiotropy tests (MR-Egger regression: *P* = 0.49; MR-PRESSO: *P* = 0.408) verified the robustness of the results (**Figure 2** and **Table 3**).

**Table 3:** MR results and sensitivity analyses between steroid hormones and DR in the discovery stage.

	*P* (MR results)					*P* (Cochrane's Q)		*P* (Pleiotropy)	
	IVW	MR-Egger	WM	MLE	MR-RAPS	IVW	MR-Egger	MR-Egger regression	MR-PRESSO
Aldo	0.07	0.97	0.60	0.02	0.02	0.23	0.21	0.49	0.408
A4	0.39	0.79	0.64	0.35	0.34	0.35	0.32	0.45	0.464
P4	0.98	0.44	0.86	0.91	0.91	0.48	0.47	0.38	0.513
17-OHP	0.73	0.75	0.37	0.53	0.53	0.31	0.24	0.82	0.386
T/E2	0.56	0.08	0.46	0.56	0.56	0.47	0.82	0.04	0.412

MR, Mendelian randomization; IVW, Inverse variance weighted; WM, Weighted median; MLE, maximum likelihood estimator; MR-RAPS, MR robust adjusted profile score; Aldo, aldosterone; A4, androstenedione; P4, progesterone; 17-OHP, hydroxyprogesterone; T/E, Testosterone_Estradiol_Ratio.

There was no significant causal association between these 4 steroid hormones (A4, P4, 17-OHP, and T/E2) and DR (all *P* > 0.05) (**Figure 2** and **Table 3**). The Cochrane’s Q test confirmed no heterogeneity in these results (*P*, 0.23 to 0.48) (**Figure 2** and **Table 3**). MR-Egger regression and MR-PRESSO test confirmed that there was no pleiotropy in these results (A4, P4, and 17-OHP: all *P* > 0.05) (**Figure 2** and **Table 3**). The results of T/E2 had pleiotropy by MR-Egger regression (*P* = 0.04), which is contrary to MR- PRESSO (*P* = 0.412). More details are shown in **Supplementary Table S2**. Additionally, the leave-one-out method was used to further validate data robustness between each steroid hormone and DR (**Supplementary Figure S2**).

### 3.3 Causal associations between steroid hormones and AMD (discovery stage)

After data harmonization of GWAS data, 18 SNPs related to Aldo, 15 SNP s related to A4, 28 SNPs related to P4, 12 SNPs related to 17-OHP, and 15 SNPs related to T/E2 were used as IVs, and the details of all IVs can be found in **Supplementary Table S1**. The 5 steroid hormones did not show a significant effect on AMD risk (all *P* > 0.05) (**Figure 2** and **Table 4**). The results of sensitivity analysis confirmed the absence of significant heterogeneity (all *P* > 0.05) and pleiotropy (all *P* > 0.05) (**Figure 2** and **Table 4**). More details are shown in **Supplementary Table S2**. Additionally, the leave-one-out method was used to further validate data robustness between each steroid hormone and AMD (**Supplementary Figure S3**).

**Table 4:** MR results and sensitivity analyses between steroid hormones and AMD in the discovery stage.

	*P* (MR results)					*P* (Cochrane's Q)		*P* (Pleiotropy)	
	IVW	MR-Egger	WM	MLE	MR-RAPS	IVW	MR-Egger	MR-Egger regression	MR-PRESSO
Aldo	0.39	0.71	0.58	0.64	0.65	0.89	0.89	0.46	0.920
A4	0.79	0.59	0.79	0.52	0.53	0.38	0.33	0.63	0.271
P4	0.82	0.70	0.45	0.94	0.94	0.18	0.15	0.74	0.196
17-OHP	0.43	0.21	0.74	0.73	0.74	0.51	0.54	0.29	0.564
T/E2	0.75	0.78	0.81	0.68	0.68	0.07	0.06	0.67	0.066

MR, Mendelian randomization; IVW, Inverse variance weighted; WM, Weighted median; MLE, maximum likelihood estimator; MR-RAPS, MR robust adjusted profile score; Aldo, aldosterone; A4, androstenedione; P4, progesterone; 17-OHP, hydroxyprogesterone; T/E, Testosterone_Estradiol_Ratio.

### 3.4 Causal associations between steroid hormones and glaucoma (replicated stage)

For the causal relationship between T/E2 and glaucoma found in the discovery stage, we verified it in the replicated stage. Based on the GWAS from the MRC-IEU consortium, eight SNPs related to Aldo, seven SNP s related to A4, six SNPs related to P4, five SNPs related to 17-OHP, and six SNPs related to T/E2 were used as IVs (**Supplementary Table S1**).

**Figure 3:**
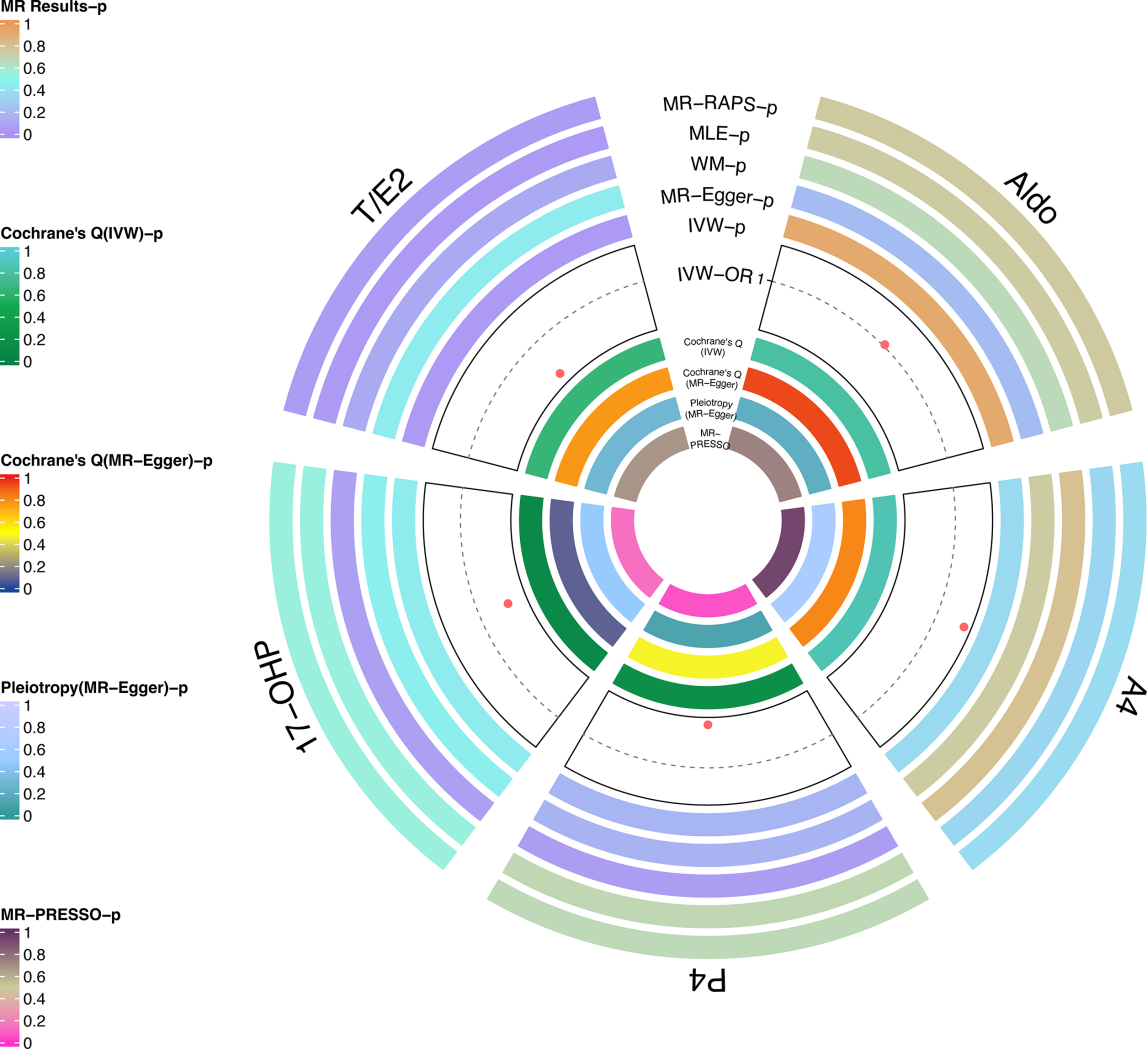
Causal analysis of steroid hormone and glaucoma based on MR analyses and sensitivity analyses in the replicated stage.

In the replicated stage, the results of IVW verified the findings at the discovery stage, that is, T/E2 was causally associated with the risk of glaucoma (*P* = 0.04), while Aldo (*P*=0.93), A4 (*P* = 0.35), P4 (*P* = 0.17) and 17-OHP (*P* = 0.43) were not significantly causally associated with glaucoma (**Figure 3** and **Table 2**). Similarly, the results of MLE showed the effect of T/E2 on glaucoma risk (*P* = 0.04) (**Figure 3**, **Supplementary Table S2**). Furthermore, MR- RAPS results showed an association between T/E2 and glaucoma that approached statistical difference (*P* = 0.05) (**Figure 3** and **Table 2**). More details are shown in **Supplementary Table S2**. At the same time, the results of Leave-on-out did not show that a single IV drove the causal relationship between each steroid hormone and glaucoma (**Supplementary Figure S4**).

## 4 Discussion

Our study systematically assessed the causal correlation of steroid hormone and RND (glaucoma, DR, and AMD) using the data from large-scale GWAS. Our study’s observational and IVW results supported the T/E2 effect on glaucoma to a certain extent, which was further validated by the WM, MLE, and MR-RAPS results. At the same time, this discovery was verified in the replicated stage. This means that glaucoma risk is affected by T and E2. The increase of the T level or the decrease of the E2 level will increase the value of T/E2, indicating that the risk of glaucoma increases. Therefore, T/E2 may be a biomarker to assess glaucoma risk. However, gene data based on a large sample did not show the effect of steroid hormones on DR and AMD risk.

Dewundara et al. reported that age at menarche and menopause, using oral contraceptives or bilateral ovariectomy would increase the glaucoma risk by reducing the duration of E2 exposure (37). In a *post hoc* analysis from a clinical trial, the intervention of E2 may become a protective factor of glaucoma by reducing IOP (38). Moreover, Nakazawa et al. showed that E2 could activate the ERK-c-Fos pathway, thereby reducing the death of RGC and exerting neuroprotective effects (39). In contrast, Estradiol regulates nitric oxide signaling and enhances ocular blood flow, thereby lowering IOP. From the genetic perspective, Magalhães et al. also mentioned that the NOS3 gene coding for endothelial nitric oxide (NO) synthase is associated with glaucoma (40). Mookherjee et al. (41) also suggested that E2 may play a role in the pathogenesis of glaucoma. When CYP1B1 is mutated, the metabolic activity of E2 may be affected, leading to the upregulation of Myocilin and affecting the development of primary open-angle glaucoma (POAG) (41). A cohort study of 63 women with POAG also found a correlation between E2 and POAG, confirming that a decreased level of E2 is a risk factor for POAG (42). Our results also verify this conclusion to some extent: reduced E2 levels increased susceptibility to glaucoma.

Notably, the control of IOP is closely related to glaucoma progress. Through pathway analysis, Youngblood et al. identified E2 as an upstream regulator of IOP-related genes such as TES (43). Jojua et al. (44) examined the levels of E2 and gonadotropins in 71 patients with POAG and the results confirmed the role of E2 and gonadotropins in IOP regulation. Therefore, we speculate that the effect of E2 on glaucoma risk may lie in the effect of E2 on IOP.

T and E2 are in dynamic balance in the body, and their imbalance may affect the risk of disease (45). This means that the effect of steroid hormones may not be limited to the change of E2. There is a gap in current research on T’s role in the glaucomatous process. A cohort study in Korea showed that the use of androgen deprivation therapy was associated with a decreased risk of POAG patients with prostate cancer (46). Bailey et al. confirmed the association between the gene variant set of T and glaucoma at the genetic level (47). Kang et al. evaluated the postmenopausal sex hormone levels of 189 POAG patients and 189 controls and found a suggestive association between elevated T levels and increased IOP and glaucoma risk (48). Our results are consistent with the above studies’ conclusions and reflect that elevated T levels will increase the risk of glaucoma. Lee et al. believed that T inhibits the activity of endothelial NO synthase in the trabecular meshwork, thereby reducing the outflow of aqueous humor and increasing IOP, which aggravates the RGCs damage. However, additional research is required to corroborate this viewpoint.

Takasago et al. administered Aldo (40, 80, or 160 μg/kg/day) to rats and found that the RGC number was significantly reduced (49). Different from the animal experiment, compared with the control group, the patients with aldosteronism have no significant difference in their prognosis of glaucomatous optic disc appearance (11). Our MR results also found no causal relationship between aldosterone and glaucoma. Due to the species differences between animal models and humans, the effects of aldosterone on the eyes are also different. Moreover, the effect of Aldo is not only in itself but, more importantly, in the Renin-Angiotensin-Aldo System’s co-regulation.

Further limitations include that our study does not support the effect of steroids on DR and AMD risk, even though we chose 1×10^-5^ (locus-wide significance) as the threshold for the IVs. Typically, a more stringent threshold (genome-wide significance threshold) should be chosen, which will further reduce the number of IVs eligible for quality control. Therefore, the effect of T/E2 on glaucoma risk still deserves to be studied in depth. In future studies, the GWAS sample size needs to be expanded to obtain more valid IVs for further validation to obtain more rigorous conclusions. Also, since there are many different types of glaucoma (e.g., POAG, primary angle closure glaucoma), we will further explore the effect of T/E2 on the risk of different types of glaucoma in the future study. On the other hand, we exclusively examined the effect of serum steroid hormones on the risk of RND. Local steroid hormone levels in the eye could be a more visual response to the effect on RND. Due to the lack of a GWAS for ocular local steroid hormone levels, further studies are needed to obtain IVs that can be used for evaluation. In addition, since the genetic variation of steroid hormones originates from the European population, the conclusions still need to be generalized with caution to extend the conclusion to Asian races.

Through MR analysis, we found a causal relationship between T/E2 and glaucoma, which provides new genetic evidence for glaucoma prevention and risk assessment. However, there is still a lack of strong evidence for the effect of steroids on DR and AMD. Further research is needed to confirm their relationship.

## Data availability statement

The original contributions presented in the study are included in the article/**Supplementary Material**. Further inquiries can be directed to the corresponding author.

## Ethics statement

To ensure that the individual information of the subject will not be disclosed, all summary-level data are from de-identified genome-wide association studies (GWASs) and can be used without restriction. All GWASs (including GWASs of steroid hormone as exposure and GWASs of RND as outcomes) were performed following the declaration of Helsinki and were approved by the ethics committee of the affiliated institution. The ethical approval for each study can be found in the original publication.

## Author contributions

KL and ZY designed the study. KL and ZY analyzed the data and drew the figures. All authors critically revised the manuscript. All authors read and approved the final manuscript.

## Supplementary material

Supplementary Figure 1 The leave-one-out results of steroid hormone for glaucoma in the discovery stage.

Supplementary Figure 2 The leave-one-out results of steroid hormone for DR in the discovery stage.

Supplementary Figure 3 The leave-one-out results of steroid hormone for AMD in the discovery stage.

Supplementary Figure 4 The leave-one-out results of steroid hormone for glaucoma in the replicated stage.
