# Supplementary figures and images for: Genetic variation reveals the influence of steroid hormones on the risk of retinal neurodegenerative diseases
Source: Front Endocrinol (Lausanne). 2023 Jan 10;13:1088557. doi: 10.3389/fendo.2022.1088557 (PMC9871487; doi:10.3389/fendo.2022.1088557)

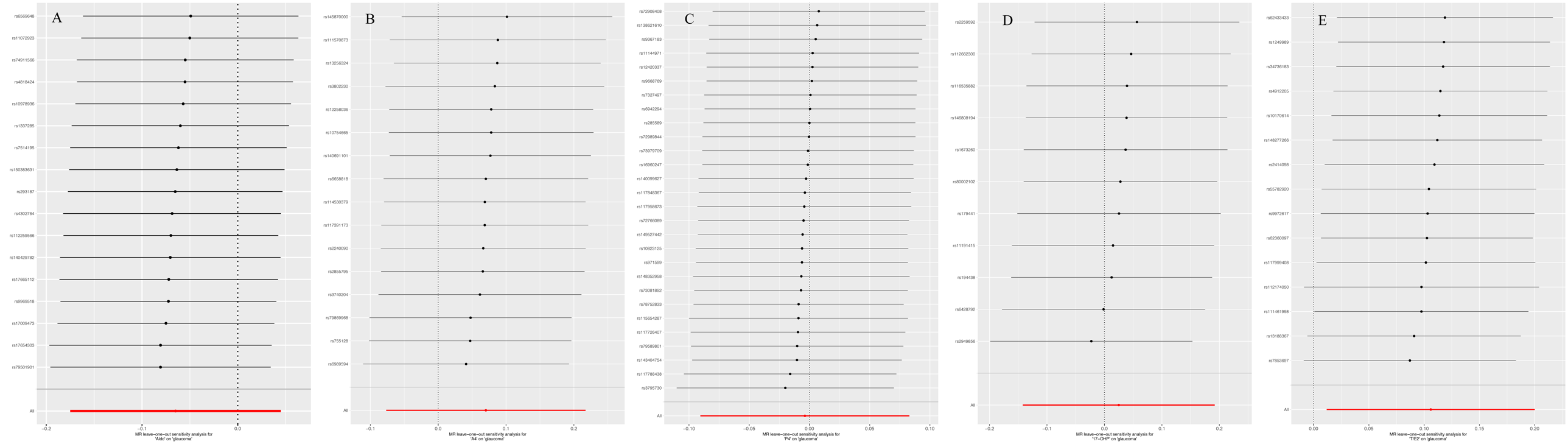

Supplement: Supplementary Figure 1 — The leave-one-out results of steroid hormone for glaucoma in the discovery stage. [file Image_1.tif]

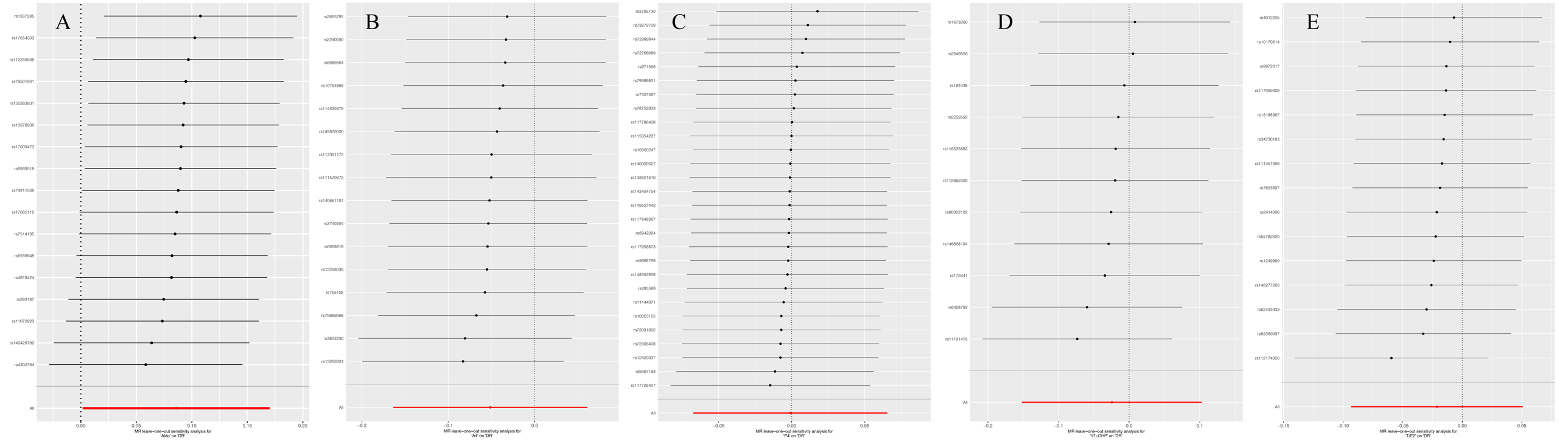

Supplement: Supplementary Figure 2 — The leave-one-out results of steroid hormone for DR in the discovery stage. [file Image_2.tif]

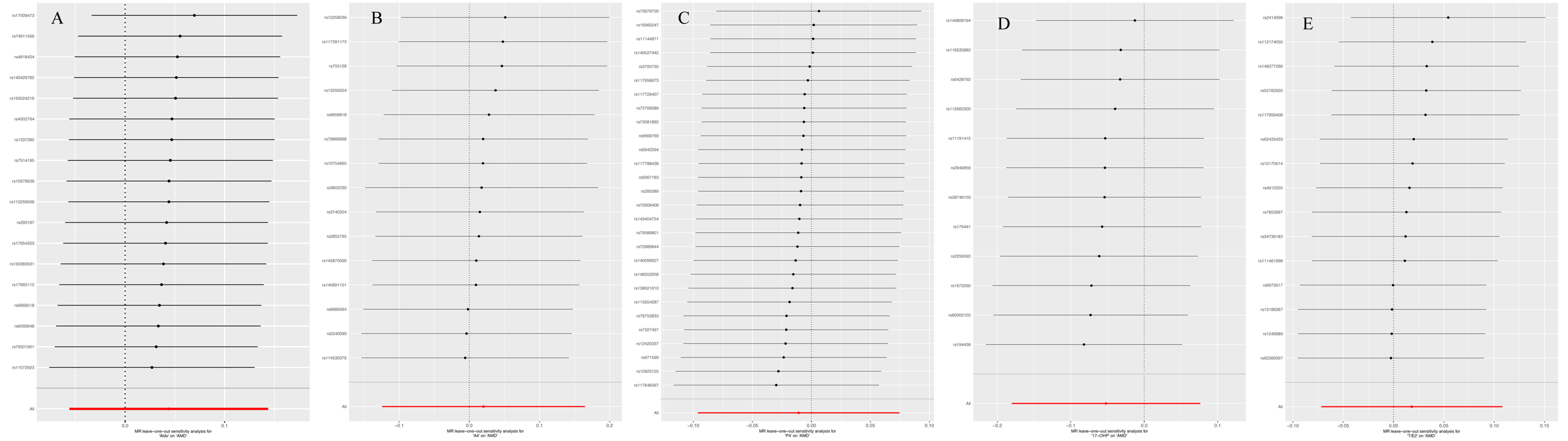

Supplement: Supplementary Figure 3 — The leave-one-out results of steroid hormone for AMD in the discovery stage. [file Image_3.tif]

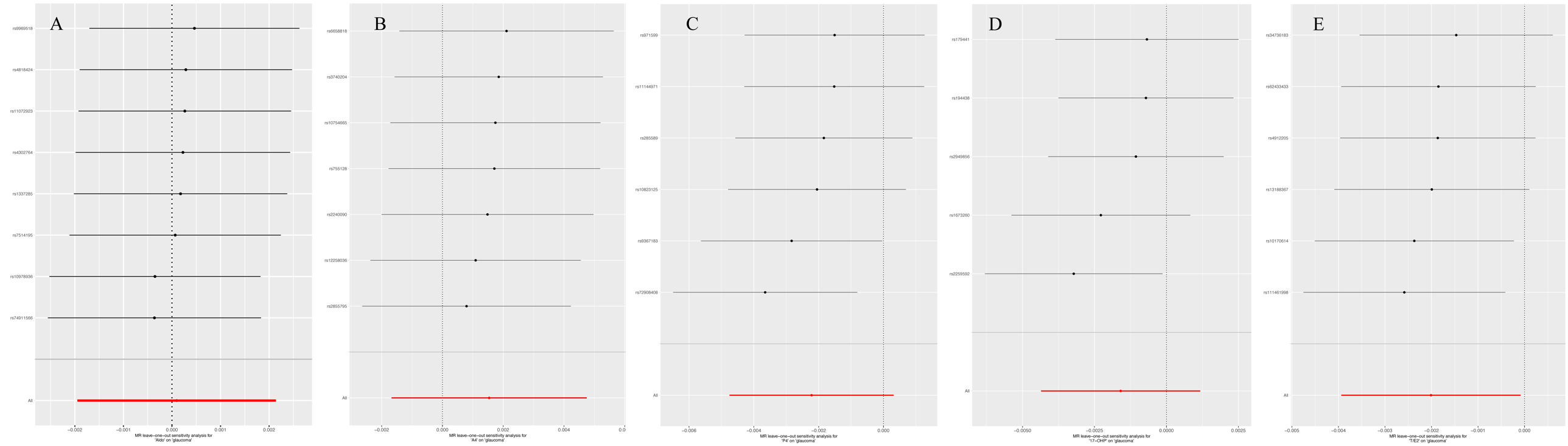

Supplement: Supplementary Figure 4 — The leave-one-out results of steroid hormone for glaucoma in the replicated stage. [file Image_4.tif]
